# Fabricating a Portable ECG Device Using AD823X Analog Front-End Microchips and Open-Source Development Validation †

**DOI:** 10.3390/s20205962

**Published:** 2020-10-21

**Authors:** Miguel Bravo-Zanoguera, Daniel Cuevas-González, Marco A. Reyna, Juan P. García-Vázquez, Roberto L. Avitia

**Affiliations:** 1 Engineering Faculty, Autonomous University of Baja California, Blvd. Benito Juárez s/n, C.P. Mexicali 21280, B.C., Mexico; cuevas.daniel@uabc.edu.mx (D.C.-G.); pablo.garcia@uabc.edu.mx (J.P.G.-V.); ravitia@uabc.edu.mx (R.L.A.); 2 Engineering Institute, Autonomous University of Baja California, Blvd. Benito Juárez s/n, C.P. Mexicali 21280, B.C., Mexico; mreyna@uabc.edu.mx

**Keywords:** electrocardiograph, AD8232, Arduino, portable ECG, AD8233

## Abstract

Relevant to mobile health, the design of a portable electrocardiograph (ECG) device using AD823X microchips as the analog front-end is presented. Starting with the evaluation board of the chip, open-source hardware and software components were integrated into a breadboard prototype. This required modifying the microchip with the breadboard-friendly Arduino Nano board in addition to a data logger and a Bluetooth breakout board. The digitized ECG signal can be transmitted by serial cable, via Bluetooth to a PC, or to an Android smartphone system for visualization. The data logging shield provides gigabytes of storage, as the signal is recorded to a microSD card adapter. A menu incorporates the device’s several operating modes. Simulation and testing assessed the system stability and performance parameters in terms of not losing any sample data throughout the length of the recording and finding the maximum sampling frequency; and validation determined and resolved problems that arose in open-source development. Ultimately, a custom printed circuit board was produced requiring advanced manufacturing options of 2.5 mils trace widths for the small package components. The fabricated device did not degrade the AD823X noise performance, and an ECG waveform with negligible distortion was obtained. The maximum number of samples/second was 2380 Hz in serial cable transmission, whereas in microSD recording mode, a continuous ECG signal for up to 36 h at 500 Hz was verified. A low-cost, high-quality portable ECG for long-term monitoring prototype that reasonably complies with electrical safety regulations and medical equipment design was realized.

## 1. Introduction

Considered one of the leading causes of death worldwide, cardiovascular diseases (CVDs) are a group of disorders of the heart and blood vessels. The World Health Organization (WHO) reports 17.7 million deaths in 2015 [1]; and the Pan American Health Organization (PAHO) indicates that CVDs cause 1.9 million deaths a year [2]. Often, CVDs result in death by heart attack or heart conditions due to lack of knowledge or identification [1]. The growing trend of home health or mobile health devices, which are medical devices for personal use, helps to address this problem because data are readily accessible through the use of technology such as smartphones, monitoring sensors, and software applications that register, transmit, and store a person’s health condition [3]. These mobile health wearables complement the equipment used in the hospital sector and extend remote care-monitoring with long-term records of the activity and vital signs while the user performs daily activities. These devices can capture events that occur infrequently or under specific circumstances and provide a realistic perspective for diagnostics. This ability can help to reduce healthcare costs, considering that it is more expensive to hospitalize a patient for days than to monitor remotely or at home to capture irregular events in cardiac activity [4,5]. Table 1 presents a list of generic ECG sensors for reference.

Developing personal medical devices is a highly relevant research field as there is a continuous need to innovate and improve healthcare. Consumers demand accurate battery-powered mobile devices to monitor continuously their health anywhere and at any time, and the mobile health wearables market is extremely fast-moving. For the successful application of personal monitoring devices, they must be small, have an appropriate signal quality, have low power requirements, be capable of long-term registration or real-time transmission, and be low cost. To meet these expectations, developers have had to find complex and costly multicomponent solutions that require high power consumption, large footprints, and long development times. Recognizing these problems, semiconductor companies have developed smaller and highly integrated chips, known as analog front-end (AFE), for more rapid designs of biopotential measurement applications. Reducing device design time is necessary to enter new markets of personal health monitors [14,15], and the use of specialized AFEs and open-source components helps to design a prototype rapidly.

**Figure 1:**
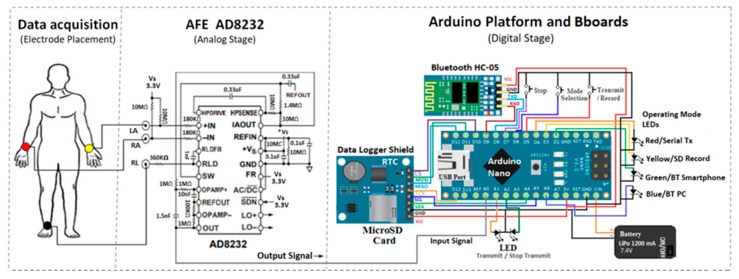
Sections of the portable ECG prototype. The circles on the person’s body represent possible positions of the electrodes. The two main components of the prototype are the AFE AD8232 and the Arduino modules (an Arduino Nano board complemented with a data logger and Bluetooth HC-05 Bboards). The Vs power supply terminal in the AFE AD8232 section is driven by the 3.3 V output of the Arduino Nano. External power is provided by a LiPo battery. This diagrammed circuit details a serial port through pins D8 and D9 of the Arduino Nano as Rx and Tx, respectively, using the SoftwareSerial library rather than the existing Arduino hardware USB port.

The objective of this study was to prototype, as rapidly as possible, a low-cost and portable single-lead electrocardiograph (Lead-I) that allows long-term recording and transmission to PC and smartphones while maintaining signal quality, as well as being developed under an open-source platform using AFE AD823X microchips. This implementation can be the base of a wearable ECG sensor or of a moderately complex and tightly regulated device, for example, of an ambulatory ECG recorder. Not only is the methodology of making the device presented, but also the functionality validation of the open-source solutions employed and improvement steps in implementing the prototype. Standard open-source components, such as Arduino shields and popular breakout boards (Bboards) designed to use I2C or SPI communication protocols that use fewer pins, produced a modular prototype with simple connections (see Figure 1). The findings obtained from using an open-source platform, such as the Arduino Nano board, which uses the Atmega328p microcontroller, are presented, highlighting advantages and disadvantages. An attempt was made to develop a functional and robust system. Following the development and testing of this modular prototype, a printed circuit board (PCB) that englobed the modules was created and evaluated.

## 2. Materials and Methods

### 2.1. Open-Source Hardware

The microcontroller system used for developing the project was a generic Arduino Nano board with the CH340 USB bus convert chip, which employs the ATmega328 microcontroller with a 16 MHz oscillator. The analog-to-digital converter (ADC) features 10-bit resolution, ±2 LSB absolute accuracy, and a sampling frequency up to 15 KSPS at maximum resolution. This sampling speed is enough for an ECG monitor application. The ADC has a separate analog supply voltage pin, AVCC, which is connected to the digital supply voltage, VCC. For the ADC voltage reference (AREF), the default configuration was used: no internal or external reference but the analog 5 Volts AVCC supply. An external precision reference or an external ADC could produce better performance, although they increase the footprint and cost, but only the validation for the default system was considered at this time, mostly to monitor the shape of the ECG waveform. Under these conditions, a 10-bit resolution (5 V/1024 = 0.00488 V of LSB) was obtained by digitizing the analog ECG signal provided by the AD8232 chip. This is a single supply design (virtual ground for the AD823X) with single-ended input channels for the ADC. The minimum analog input value represents GND and, when the input is equal to AVCC, the software function analogRead() returns the value 1023. Midsupply represents approximately the baseline reference level.

Other modules or open access breakout boards were used, such as the HC-05 Bluetooth module and a data logger shield. The HC-05 module allows the wireless connectivity of the device with a friendly programming interface, using AT modem commands (attention commands) and a maximum speed of 115,200 bauds, which was imposed by the smartphones tested. The data logger shield incorporates a real-time clock, with a resolution of one second and date registration, and adds a microSD card for the physical storage of up to 32 gigabytes, allowing extended registration time. The hardware compatibility and functionality of these boards were evaluated for the acquisition, transmission, and recording of data to develop a portable ECG for long-term monitoring.

Regarding ECG signal conditioning, the EVAL-AD8232 board from Analog Devices was initially used. This board incorporates the AD8232 heartrate monitor front-end microchip conveniently mounted with test pins, switches, and jumpers to configure the circuit for application evaluations. The electrodes used were Ag/AgCl from Kendal, model Coviden Medi-trace 200, with dimensions 3.6 cm diameter. Once a specific AD8232 circuit configuration was selected, the prototype breadboard of Figure 1 was implemented with the open-source hardware elements. Table 2 details the connections between the boards and components of the ECG prototype of Figure 1. This implementation also accepts the AD8233 AFE device, which has a smaller package and several electrical specification improvements compared with the AD8232 [16].

### 2.2. Open-Source Software

The open-source software used to develop the project’s programming was the Arduino integrated development environment (IDE), with its add-ons of Serial Monitor, COM ports, and Serial Plotter. Libraries included in the Arduino IDE were used, such as the SD library for writing to a microSD, and the Serial library to transmit to a PC using the UART port via a USB cable (Rx and Tx pins). Other external libraries, such as SoftwareSerial, were used to create or simulate a UART port for communication with the Bluetooth module (pins D8 and D9 were configured) that does not interfere with the USB serial communication of the Arduino IDE, which uses the hardware ports of Rx and Tx to upload new sketches. The TimerOne library [17] was used to program an interrupt for the sample rate of analog data with a default configuration of the ADC. It was decided to use the interrupt technique to capture the ADC inputs because using the polling method to determine the ADC status generates delays and can waste important resources (memory and processing). By using interrupts, a more efficient process is achieved, which allows additional functions, such as digital filtering, to be explored. A circular buffer example was implemented for digital filtering to eliminate noise (see Section 2.5 for description). The RTClib library [18] from Adafruit, a Bboards development company, was used to configure and manage the real-time clock of the data logger shield. The library contains the date record format of years, months, days, hours, minutes, and seconds. These libraries are available in the GitHub open-source repositories. To use a generic Arduino board with the CH340 USB bus convert chip, it is necessary to install a driver to work with the Arduino IDE, which, depending on your operating system, can be installed automatically or not. Other open-source software, such as Theremino, a visualization software for ECG files on grid paper [19], and the Android Bluetooth Graphics application for real-time display of the ECG signal, were also used.

### 2.3. Analog Filter Stages and Circuit Simulation

**Figure 2:**
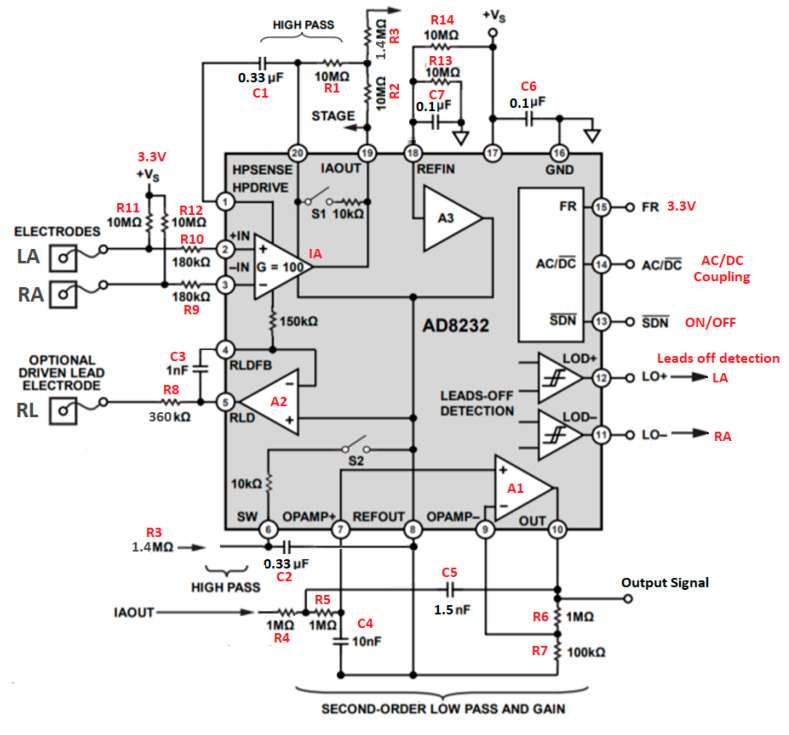
Analog Device’s AD8232 simplified internal structure and the cardiac monitor configuration. LA, RA, and RL correspond to the electrodes; R11 and R12 to polarization resistors; R10 and R9 to resistors to protect the user in case of failure; R8 and C3 are protection to limit leakage currents <10 uA; R4, R5, R6, R7, C4, and C5 represent the section of resistors and capacitors for low-pass filter; R6 and R7 represent resistors that determine the total gain of the circuit; C1, C2, R1, and R2 are resistors and capacitors that define the cutoff frequency of the high-pass filter; R3 (or Rcomp) for the filter response curve; R13 and R14 are voltage divider resistors for the midsupply reference; and C6 and C7 are noise filters. The image was modified from the source [24], Copyright © 2019, Analog Devices, Inc. All Rights Reserved.

Belonging to the family of AFE highly integrated circuits for the acquisition and conditioning of biological signals, AD823X microchips provide the analog ECG output in this project’s application. Some semiconductor manufacturers of AFEs, such as IMEC, Texas Instruments, and Analog Devices, offer this type of analog microchip to reduce space and energy consumption and to miniaturize designs for portable applications. Literature related to ECG design and the applications of the AD8232 chip [20,21,22] was reviewed. The AD823X manufacturer presents three application configurations in the datasheet, in which it states the bandwidth through high-pass and low-pass filter, with their respective equations to calculate cutoff frequency and gain. We tested the “cardiac monitor” application from those presented in the datasheet [23]. This configuration presents maximum passband flatness designed for monitoring the shape of the ECG waveform; therefore, the filter stages were set accordingly: overall gain of 1100 × and a bandwidth of 0.5–40 Hz. Each circuit section corresponding to the filter’s function and gain is explained next. Figure 2 illustrates the simplified internal structure of the AD8232 microchip, in which the instrumentation amplifier (IA) block includes a dc-blocking amplifier (high-pass filter) and provides a fixed gain of 100 to the system. Amplifier A1 is used for the low-pass filter and the variable gain stage of the circuit and was calculated using the equations in the datasheet. The A2 amplifier is used for right foot reference and common mode injection to the system improving the CMRR of the IA.

Other ECG applications (chest measurement or fitness monitoring) suggest modifying the cutoff frequency of the high-pass filter to 7 Hz to reduce noise from motion artifacts, but this can modify the ECG waveform. To obtain an ECG waveform with minimal distortion at resting position, the AD8232 was configured with a 0.5 Hz two-pole high-pass filter and a 40 Hz two-pole low-pass filter (cardiac monitor configuration). Resistors R4 and R5 and the capacitors C4 and C5 correspond to the low-pass filter and, by modifying these values, the cutoff frequency (fc) is modified. Equation (1) corresponds to the cutoff frequency of the low-pass filter for the circuit configuration of Figure 2.

(1) $$\mathrm{fc}\;=\;\frac{1}{(2\pi \surd \left(R4\;C5\;R5\;C4\right)}$$

Using the values R4 = R5 = 1 MΩ, C5 = 1.5 nF, and C4 = 10 nF, the cutoff frequency of the low-pass filter is 41.09 Hz. The resistors R7 and R6 correspond to the gain and can be modified with the following equation:

(2) $$\mathrm{Gain}\;=\;1+\left(\frac{R6}{R7}\right)$$

Using the values R7 = 100 KΩ, and R6 = 1 MΩ, the gain of this stage is 11, and when multiplied by the fixed gain of the IA, the total gain is 1100. The capacitors C1 and C2 and the resistors R1 and R2 correspond to the high-pass filter. By modifying these values, the cutoff frequency (fc) is set in accordance with Equation (3).

(3) $$\mathrm{fc}\;=\frac{10}{2\pi \;\sqrt{R1}\;C1\;R2\;C2}$$

By applying the values R1 = R2 = 10 MΩ, and C1 = C2 = 0.33 µF to the Equation (3), the cutoff frequency is 0.48 Hz. The resistance R3 should be 0.14 times the value of R1 and R2 to optimize the filter for a maximally flat pass band; lowering the R3 value can increase the Q and, consequently, the peak of the filter. Other features, such as the third electrode driver, input bias resistors, fast restore function to reduce the stabilization time, and the alerts in the event that any of the electrodes is disconnected, are explained in the AD823X chips documentation [16,23].

**Figure 3:**
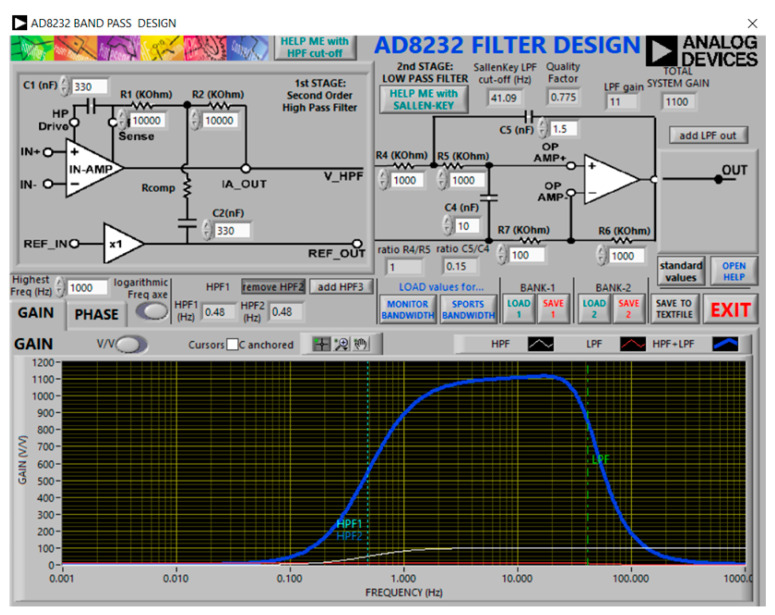
Cardiac monitor ECG circuit and response using Filter Design software [25], Copyright © 2019, Analog Devices, Inc. All Rights Reserved.

The manufacturer provides software called AD8232 Filter Design [25] (see Figure 3), which allows one to set the value of the components and to see the filters responses in magnitude and phase, and cutoff frequencies. We used this software to check the consistency of the bandwidth and formula calculations. The filter design software separates the design in two stages: a high-pass filter (first stage) and a low-pass filter (second stage). The first stage has the option to use three high-pass filter templates, HPF1, HPF2, HPF3 tabs, as displayed in Figure 3. The HPF2 option was activated for our application, and the resistance values must be those indicated in the figure to have an fc = 0.48 Hz in the high-pass filter. In the low-pass filter, the “add LPF out” tab must be deactivated and, with the indicated values of resistors and capacitors, the value of the cutoff frequency is fc = 41.09 Hz, with a total gain of 1100 and a Q = 0.775. The software has filters for pre-designed applications, but in our case the values for the cardiac monitor application from the data sheet of the AD8232 chip were used, and are displayed in Figure 2 and Figure 3. Furthermore, the software displays the representation of the responses and the bandwidth for this circuit, and the cutoff frequencies corresponding to those calculated.

Prior to the prototype construction, Multisim SPICE was employed to simulate the circuit from a macromodel provided by Analog Devices [26] with the ECG generator tool from LabVIEW to create ECG signal data. To simulate the circuit under different critical operating conditions, the stability of the system was assessed using several methods: Monte Carlo, Fourier analysis, worst-case, and temperature variation.

### 2.4. AFE AD8232 and AD8233, Adapters for Breadboarding and PCB

**Figure 4:**
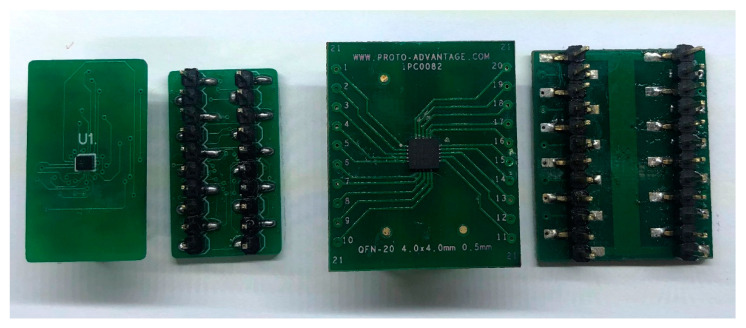
SMD-DIP (breadboard) Adapter for AD8232 and AD8233 microchips.

To implement these microchips, an SMD-DIP adapter was used to assemble a breadboard prototype circuit and then to devise a design on a custom PCB. As an alternative to acquiring this type of adapter, the company Proto-Advantage offers SMD-DIP adapters for a wide variety of SMD components [27], including one for the AD8232. However, a custom SMD-DIP adapter had to be implemented, since no commercial version was found for the AD8233 chip; Figure 2 presents the two versions of the adapter. As space-saving and improved electrical performance are features of the new AFE AD8233, its small package connects to a PCB using solder balls with a pitch of 0.4 mm; thus, the standard trace width of 5 mils cannot fit between its pins [28]. Therefore, board fabrication becomes more difficult and costly as it requires smaller-sized tracks and vias than the standard used by most manufacturers. The advanced manufacturing option allows trace widths as narrow as 2 mils. The custom SMD-DIP adapter was built in a dual-layer board with 2.5 mils trace widths (advanced capabilities) and standard 23.6 mils vias, as displayed in Figure 4. The manufacturing cost of these small tracks was lower than a via-in-pad version.

**Figure 5:**
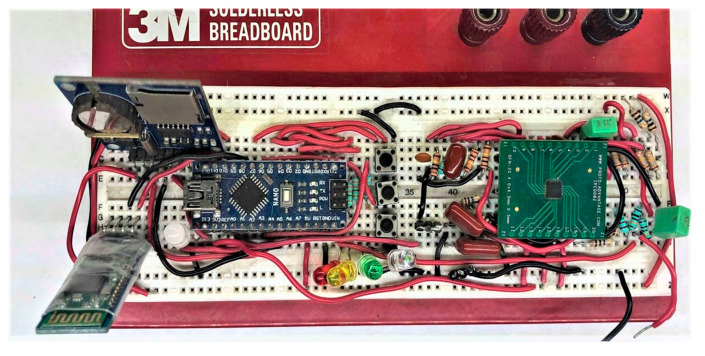
ECG monitor breadboard circuit with interchangeable AD8232 and AD8233 microchip adapters.

To produce a modular prototype with standard digital connections, popular Bboards were used. The compatibility of Arduino and its communication protocols SPI, I^2^C, and UART were considered to reduce the number of connections for a portable design. Initially, the operation, range, and connectivity were evaluated by breadboard wiring the circuit in Figure 1, as displayed in Figure 5, and uploading with code for serial transmission, microSD recording with time and date, and Bluetooth transmission. A menu was developed to mix the signal acquisition step with four possible operating modes.

**Figure 6:**
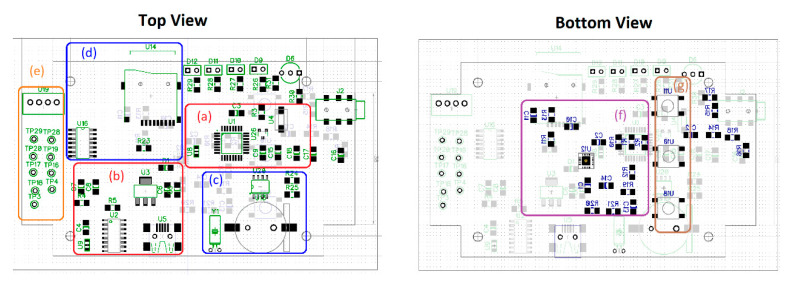
Top and bottom view of the printed circuit, highlighting distinct areas. (**a**,**b**) components of the Arduino Nano board; (**c**,**d**) data logger shield; (**e**) HC-05 Bluetooth module terminals and Vout, Vin, GND pins; (**f**) ECG circuit with AD8232 microchip; and (**g**) push-button peripherals.

Following the construction and testing of the prototype breadboard, a printed circuit (custom PCB) was manufactured with the simplified design of the generic Arduino Nano CH340 board. The basic components for its operation were selected: the ATmega328 microcontroller, the CH340 USB bus convert chip, an LM1117 voltage regulator, a reset button, oscillators, and a mini-USB socket. The microcontroller was used with the factory-preloaded bootloader for the Arduino IDE and its USB connectivity, but the use of ICSP communication was precluded. Therefore, although a program (sketch) can be modified or updated, it was not possible to modify the factory bootloader. The essential elements of the data logger module were used: the DS1307 RTC, a logic level regulator from 5 V to 3.3 V, a crystal, a coin battery socket, and a microSD card reader. The HC-05 Bluetooth module was used without modifications as it includes the necessary elements for wireless transmission (antenna and RF transceivers). The DipTrace’s schematic capture and PCB design software were used to create a double-sided board (element layout illustrated in Figure 6), generating the Gerber RS-274X files for PCB manufacturer services. The PCB capabilities, specifications, and limitations of the manufacturer were loaded so the software’s design rules verification process automatically marked errors for non-compliance. Precision components with 1% tolerance were used, which conforms to the values established in the regulation standards of the IEC 60601-1-9, ANSI/AAMI/IEC 60601-2-47:2012 [29,30]. The printed circuit uses SMD 1205, SO4, SOT223, and SOT23 component footprints. For the single integrated board design for this prototype, duplicated components appearing in the breadboard version were eliminated: 3.3 v regulators, an LED, two resistors, three capacitors, and a diode. Furthermore, the resistance values of the LEDs were adjusted to reduce consumption and increase battery life. The total breadboard current consumption in microSD recording was 126 mA and, after component reduction, the current fell to 68 mA, which corresponds to a 46% energy saving. Only the AD8232 PCB version was manufactured and tested.

### 2.5. Data Output Modes

The data collected from the ECG signal can be used in four ways: (1) serial cable transmission, (2) microSD memory recording, (3) Bluetooth transmission to a smartphone, and (4) Bluetooth transmission to a PC. Common to all these operating modes is the signal acquisition step. Online display is realizable for Operating Modes 1, 3, and 4, and offline display for Operating Mode 2. In serial transmission (Operating Mode 1), data are sent in real-time for visualization in the Arduino IDE and in the LabVIEW environment. In microSD memory recording, Operating Mode 2, data are stored for subsequent processing or visualization in tabulated form, compatible with software such as Excel, MATLAB, and Theremino. In Mode 3, the data format of the Bluetooth transmission to a smartphone is compatible with the Bluetooth Graphics application for data display on a smartphone or tablet with the Android system. In Mode 4, the Bluetooth transmission to a PC is configured to display data in both the Arduino IDE software and the LabVIEW environments. All operating modes, except the microSD memory recording, require an external device for graphic visualization or monitoring. Moreover, while streaming allows real-time visualization, storage is limited to the data buffer size for pixel display or of the IDE Serial Monitor in these modes. Only SD card recording provides deep data storage.

**Figure 7:**
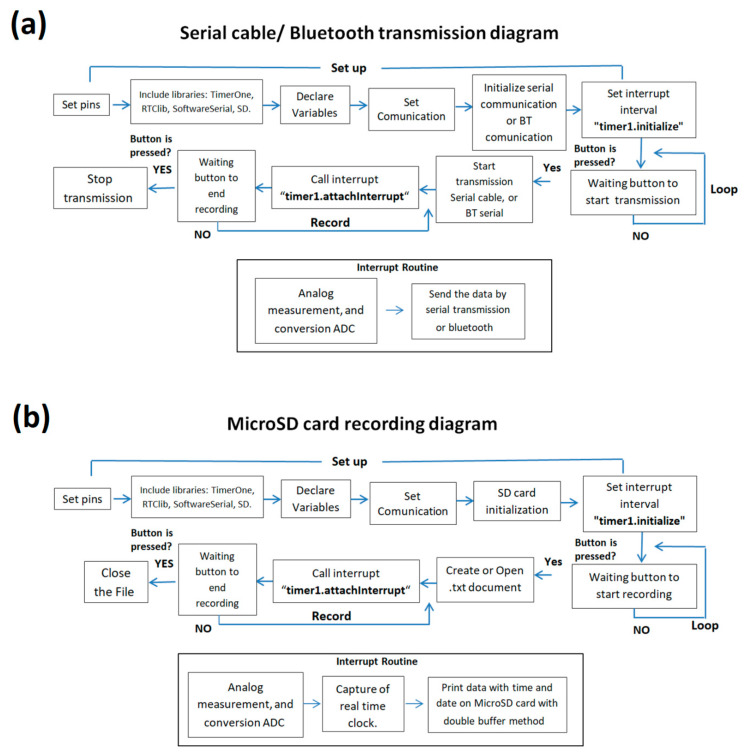
(**a**) Serial cable/Bluetooth transmission program diagram; (**b**) MicroSD card recording program diagram.

Once a link is established in the serial transmission operating modes (of 1, 3, and 4), sample data are transmitted as soon as they are acquired. In Mode 2, MicroSD recording, a packet is built with the double buffer method explained in Section 2.7. Figure 7 contains program-flow diagrams for the two types of transmission/recording process. The efficiency of the processes is related to the maximum sampling frequency of the analog signal to produce a transmitted/recorded data without presenting errors, from the data acquisition to data display or storage. This relationship is supported because, in the interrupt service routine resides the transmission of data to the external device.

**Figure 8:**
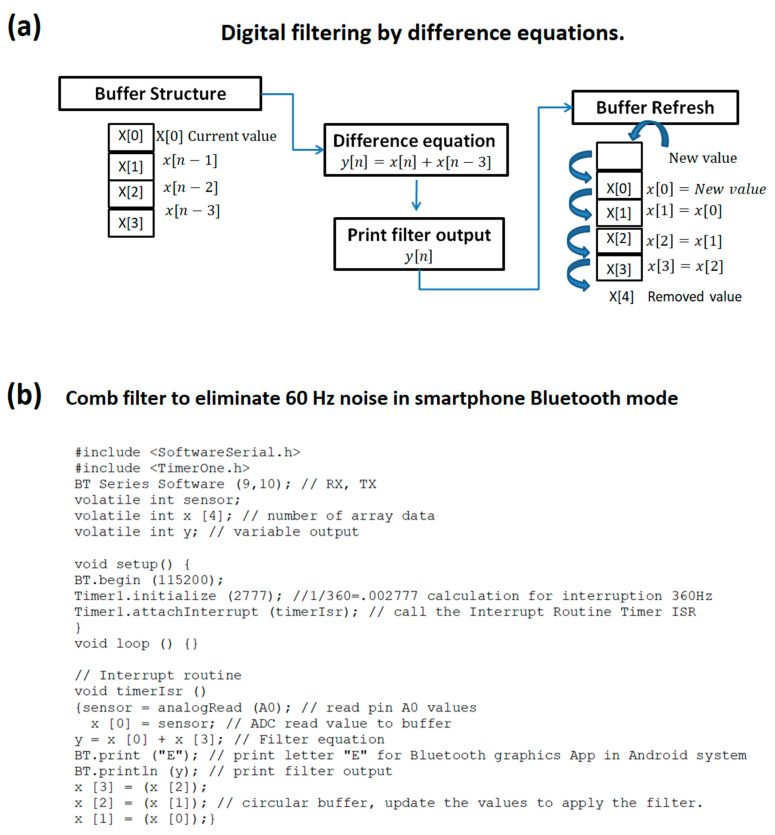
(**a**) Circular buffer for digital filter y[n] = x[n] + x [n − 3]; (**b**) code to implement the digital filter with difference equation on the Arduino platform, Bluetooth transmission, and smartphone visualization.

In addition to data acquisition, recording, and visualization (operating modes), the system was tested with a signal processing load: a basic circular buffer was implemented for digital filtering using short difference equations to eliminate noise [31,32]. With a sampling rate of 360 Hz, the idea was to eliminate 60 Hz noise and harmonic components. Figure 8 is a diagram of a circular buffer to apply the difference equations and the implemented code to transmit by Bluetooth on Arduino to smartphone.

### 2.6. Hardware/Software Testing

The necessary tests were performed to evaluate the hardware and software to ensure the functionality and robustness of the prototype. To test the ADC in the Atmega328 microcontroller, adjustable voltage sources were applied at the input, and the digital output measured and compared with the applied voltages. Using the adjustable voltage source from Digilent’s Analog Discovery 2 instrumentation system, the test included variation of the ADC input voltage from 0 V to 5 V in incremental steps of 0.5 V.

To check the AD8232 functionality and stability, ECG signals were acquired from a simulator and real patients. Simulated ECG signal parameters were initially tested using the SimCube SC-5 simulator [33] from Pronk technologies. Additionally, ECG signals were obtained from a healthy patient using the breadboard prototype and also with the PCB version to observe the influence of the different components on the signal quality of real ECGs. Baseline noise was measured on smooth segments of the ECG as a simple characterization of noise during real operation. Furthermore, the measurement of the output noise when the input is zero (electrode inputs tied together) provides a performance metric. In addition to these quantitative measures, a qualitative visual comparison was achieved using a medical desktop ECG (model mitral M12USB from Korell [34]) of a human volunteer and the one obtained using our fabricated system. On regular basis, a qualitative visual baseline comparison of all the plots was carried out.

Once the function of the AD8232 chip was verified, the digital part of the prototype (Arduino Nano and Bboards) was also evaluated but with virtual ECG signals. The virtual signals were created using the ECGSIM function of MATLAB [31], which generates a synthetic ECG signal. This synthetic ECG signal was used in two types of tests: (1) To verify the stability of the ADC with recording operation, using a programmable function generator such as Digilent’s Analog Discovery or Tektronix AFG3021B to reproduce this signal cyclically to serve as an input ECG signal to convert and record in microSD in long-term registers (up to 36 h); (2) The virtual signal was also used to create ECG control signals to evaluate automatic analysis algorithms for data loss prevention, as described in Section 2.7 and Section 2.8.

To characterize fully the microcontroller and peripherals response, maximum sampling frequency validation tests were performed in each operating mode. To measure the data transferred per second for the serial PC, Bluetooth smartphone, and Bluetooth PC operating modes, a timing technique was used with Arduino´s millis() function: by starting a time flag before the reading by the ADC, and placing another flag when printing the data, an elapsed time can be calculated from the printouts. In microSD card mode, it was decided to use the RTC data register as it is in its actual operation to calculate the data rate from the printouts. In both cases, a function generator with the created ECG signal was used as the input signal. Using interrupts, an initial sample rate was established at 500 Hz to capture the analog ECG signal and, supported by the Excel software, the transferred data and time records were counted to validate the information. For the maximum sampling frequency, the time between each interruption was decreased to increase the sample rate until reaching a value at which errors or loss of information occurred when verifying the transmission of data in Excel. In this way, the limits at which the system could reliably transfer data were established.

For current consumption, a Fluke 111true RMS multimeter between the prototype and the LIPO 7.4 V battery was used. A leakage current meter of type UNI-T UT251A, which has a sensitivity of 1 µA, was utilized on the leg electrode. Basic safety and essential performance were assessed for comparison of the prototype parameters with the regulatory requirements of the ANSI/AAMI/IEC 60601-2-47:2012.

### 2.7. Buffer Overrun 

**Figure 9:**
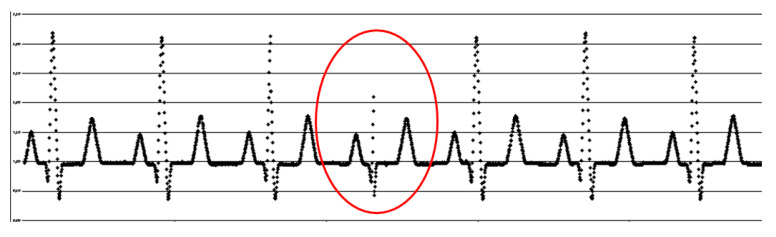
Blackout event in a synthetic ECG signal. The input signal to the system for this figure was created using MATLAB software.

Using open-source software code facilitates function development, but there are limitations. The design cannot be considered sufficiently robust for application in a real-world environment. The developer must validate, correct, and improve possible failures or limitations that may arise in open-source software. One such issue was the occasional loss of a chain of data samples during the microSD recording mode. When recording for periods longer than one hour, about 20 run samples were randomly lost (as illustrated in Figure 9). These blackout events were identified as occurring in the QRS complex.

The blackout events occurring in some of the 1 and 2 h recordings were characterized to ascertain their origin and to improve detection. To determine the origin of the problem, an experimental design was performed to exclude the influence of the following factors:

- Function generator produces the ECG signal (recordings were made using Digilent’s Analog Discovery and Tektronix AFG3021B function generators).
- Error due to the Arduino module (recordings were made using the Nano, Mega, and Uno models).
- Hardware error in the data logger module (tests were made using two different data logger models).
- SD card memory error (class 2, 4, and 10 memory cards were used).

**Figure 10:**
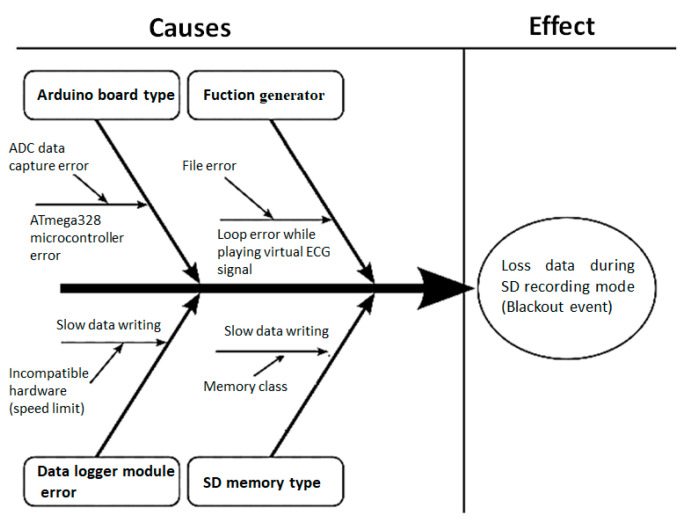
Ishikawa diagram to identify causes of the problem. Identified problem: loss of information with the occurrence of blackout events during writing to microSD. Possible causes: Arduino type, function generator, error by data logger module, or SD card memory type.

A design of experiments methodology was implemented to identify the cause of the blackout problem considering the possible factors that influence. A frequently used tool is the Ishikawa diagram, in which the possible factors or causes that originate or affect the output variable and generate the problem are identified. The blackout event effect was analyzed using an Ishikawa diagram (see Figure 10) to identify its factors and their possible influence.

**Figure 11:**
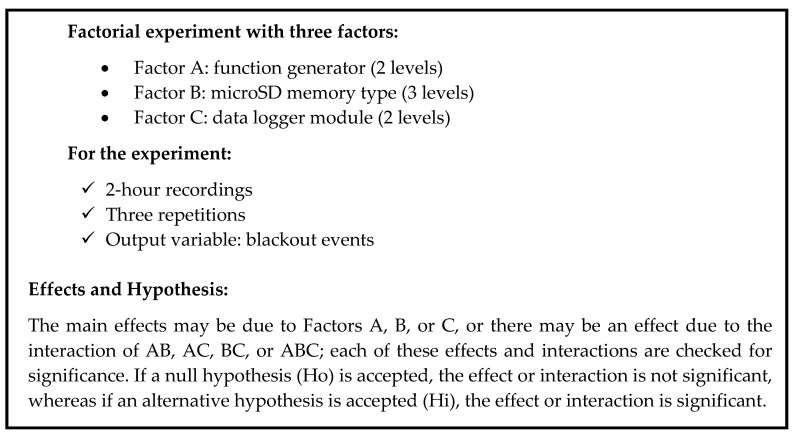
Structure, hypothesis, and effects of experimental design.

To exclude an error due to the Arduino board model, recordings were made using the Uno, Nano, and Mega models. No variation was found in the occurrence of blackout events due to this factor; thus, the design of the experiments focused on the other three variables. Once the problem was identified and the three possible variables were defined, these data were used to design a factorial experiment (see Figure 11 and Table 3).

To execute the experimental design, it was defined as a run: an ECG signal recording in the microSD mode with a duration of two hours; and as an output variable: the number of blackout events present in the recording. The order of the runs should be randomized to minimize equipment or environmental errors; thus, a function of the Minitab 15 software was used that randomly orders the runs. The Minitab software does not analyze the ECG signal registers; it was necessary to verify independently and count the blackouts and enter the number of blackout events in the Minitab 15 software. This study reveals the factors or the relationship between them that significantly influence the occurrence of blackout events. Offline algorithms for the automatic detection of QRS complexes in ECG signals were implemented to detect blackout events in high-volume data streams, as explained in Section 2.8. Based on the Table 3 experiment structure, Table 4 details the number of blackout events identified in the different runs according to the factors of interest.

Another tool used in the design of experiments is the analysis of variance (ANOVA). This analysis is a parametric statistical technique for hypothesis testing that reveals how one or more factors influence the mean of a variable. This technique was used to determine which of the three factors named in Figure 11 influence the appearance of blackout events. After experimenting and collecting the number of blackout events with combinations between factors, an ANOVA was used to determine, based on the number of blackout events, which of the factors or their interactions are significant or affect data loss. Table 5 presents the ANOVA report, in which a *p*-value < 0.05 means that the factor or interaction has a significant effect on the output variable. Only Factor B has a significant effect on the occurrence of blackout events. This factor corresponds to Classes 2, 4, and 10 of microSD memory cards used during the recordings. This characteristic of microSD memory cards corresponds to the speed of writing and reading. In Memory Class 2, the latency time is greater than in Classes 4 or 10. As illustrated in Table 4, the rows of Class 2 (Factor B) contain a greater number of blackout events, regardless of the other factors.

**Figure 12:**
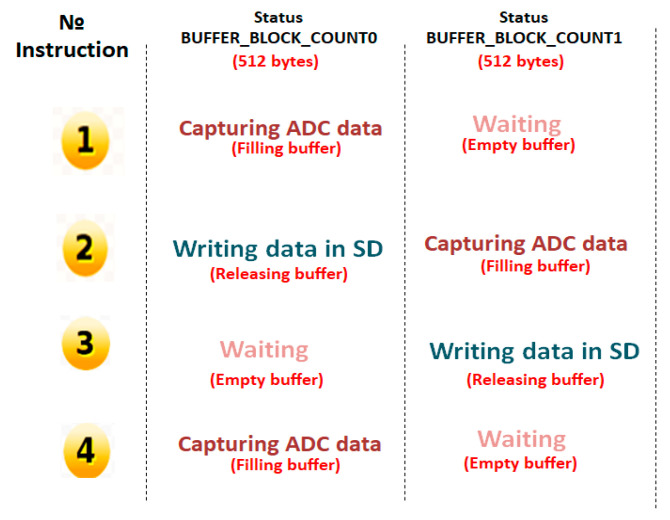
Double buffer implementation for writing data to SD memory.

The design of the experiments ascertained that the write latency in microSD memory cards influences the occurrence of blackout events. Based on these ANOVA results, the troubleshooting steps were to analyze the data transfer methods during Mode 2, microSD recording, and to create a program to measure the write latency of the different classes of microSD memory. These steps revealed that the conventional Arduino libraries work with one buffer for writing to the microSD and, due to the write latency, the buffer was overrun. To fix this problem, a secondary buffer was implemented using the microcontroller’s RAM to avoid saturation [35]. Figure 12 displays the double buffer implementation for writing data to the SD card. When initializing the SD card mode, the buffer_count_0 is populated with data from the ADC while the buffer_block_count1 waits; in Instruction 2, the full buffer (buffer_block_count0) writes the data to the SD memory while the empty buffer (buffer_block_count1) begins to fill; in Instruction 3, the full buffer (buffer_block_count1) writes the data to the SD memory while the buffer_block_count0 is waiting; and finally, in Instruction 4, the state of both buffers repeats those of the initial instruction in the write cycle until the end of the SD card mode. The best offline algorithms for the automatic analysis of ECG were then used to verify that the flaw was corrected satisfactorily.

### 2.8. Offline QRS Detection Algorithms for the Automatic Analysis of ECG Signal Recordings

Detecting QRS events manually is time-consuming, tedious, and prone to visual error. Therefore, using automatic analysis algorithms for ECG signals was proposed. Blackout events were detected in the QRS complex, so algorithms were selected that can count R peaks (see Table 6) in a large volume of data. To select the best algorithm, an ECG signal created using MATLAB software was used, corresponding to 1 and 2 h recordings (with their corresponding QRS), and this signal was modified with five blackout event cycles to produce a known virtual ECG signal in which algorithms were used for detection. Table 7 lists the algorithm evaluation to detect the best algorithm for automatic ECG signal analysis. Algorithms that had errors in counting the number of R peaks in the known signal were discarded. The results indicate that the most accurate algorithms are Pan-Tompkins and Biomedical Workbench, as these two methods provided 100% recognition. Then, these accurate algorithms were used to assess the quality of the ECG recordings during the subsequent experiments, including the experiments performed to correct for the data loss.

## 3. Results and Discussion

The prototype developed is a single-lead electrocardiograph with a bandwidth of 0.5–40 Hz, gain of 1100×, CMRR of 88.78 dB, and a patient leakage current of 1–2 µA. The prototype allows an electrode operating offset of ±300 mV, and an operating differential input range of 3 mV without saturating the signal of interest. The microSD card has a current consumption of 27 mA in standby mode and 68 mA in recording mode. Table 8 lists the breadboard prototype specifications: the bandwidth was implemented for the monitoring application stated in the ANSI/AAMI/IEC 60601-2-47:2012 standard. The sampling frequency exceeds the minimum required for this application. The dynamic range of 3 mV is insufficient for the specification established in the regulations, since the design of cardiac monitor implemented was proposed in the AD8232 datasheet (gain = 1100, for rest condition) and did not contemplate complying with the 10 mV of the normative. However, the modification necessary to meet this value specification is straightforward: the system gain must be lowered to 330 or less (see Equation (2) in Section 2.3). The leakage current was measured using a leakage current meter model UT251A and is less than allowed to guarantee the safety of the user. The CMRR is above the required value. The input impedance and DC offset were obtained from the AD8232 microchip datasheet and meet the requirements. Regarding noise specification, it was measured at the analog output of the AD8232 microchip and at the digital output and found to be very close to that stated in the microchip datasheet and was also below the maximum permitted. The precision of the passive components also meets the requirements of ANSI/AAMI/IEC 60601-2-47:2012. The filter design software provided by the manufacturer, to use with the AD823X microchips, resulted in a useful tool to set the response of the filters, gain, and cutoff frequencies, simplifying the analog filter design process. The results obtained in the simulation of the analog circuit in Multisim, from the SPICE macro model, indicate the recovery of the ECG signal with standard morphology under different critical operating conditions. With 1% tolerance values for the electronic components of resistance (Ω) and capacitance (F), as indicated by the regulations, there was no significant change in the signal output, nor did it vary the desired amplification or noise. Finally, a simulated temperature sweep of 0 °C–70 °C revealed no compromise of the signal.

From the test of applied voltages to the ADC input, the measured digital output voltages were off by less than 3% in the worst case, or a maximum error of 2 LSB in some readings. The standard deviation of 1000 readings associated to each fixed input voltage was always within ±1 LSB. All these tests were applied to the breadboard prototype; therefore, we expect similar performance or an improvement for the PCB version. A drawback in the design was the mismatch between the AD328X signal output swing and the input voltage range of the ADC. This match couldn’t be achieved at this time, because the AFE Vs supply is 3.3 V while the VACC of the ADC and its AREF were equal to the 5 V of the ATmega328 VCC (default Arduino Nano wiring). This leaves wasted conversion range, which could be fixed when AREF of the ADC independently connects to the Vs supply of the AFE microchip. Furthermore, if an ADC precision voltage reference is considered, even more accuracy would be obtained.

The different operating modes include serial communication, SD card recording, and Bluetooth transmission (smartphone and PC). The amplitude and duration of the waves and segments of the ECG signal obtained were measured to corroborate the ECG standard. The system total latency depends on two factors: (1) the baud rate communication established or clock in SDcard mode, and (2) the interruption interval established. As displayed in Table 9, for a higher baud rate more data can be sent, without exceeding the limit established in the table. Moreover, the minimum latency for transmission of data is the minimum interrupt interval in which the system does not present loss of information. To avoid losing information in each transmission mode, maximum sampling rates and programmed interruption intervals were used (see Table 9). One of the limitations of the prototype is that its maximum sampling rate decreases for wireless transmission to 909 samples per second, although this speed is sufficient for ECG applications. The prototype can sample up to 2380 Hz (samples/s) without the loss of data in serial cable transmission. 

**Figure 13:**
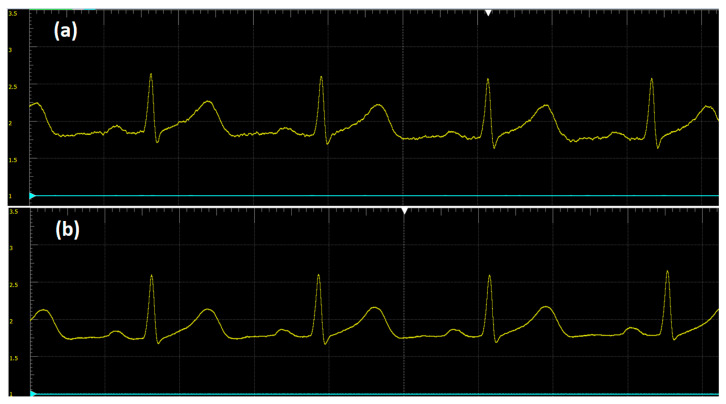
Comparison between AD8232 and AD8233 on a breadboard circuit using a male volunteer ECG signal. (**a**) Signal obtained from the AD8232 microchip on a breadboard circuit. (**b**) Signal obtained from the AD8233 microchip on a breadboard circuit. Oscilloscope hardware: 14-bit Analog Discovery with 500 mV/div amplitude and 290 ms/div in time base; software is Waveforms. Observe the improvement for the AD8233 output signal in (**b**).

Also evaluated in the development of the prototype were the AFE AD8232 and AD8233 microchips; the latter was developed by the semiconductor company to improve the structure of its AD8232 version. Figure 13 presents a human volunteer ECG signal obtained from the AD823X chips on a breadboard, and there is a visible improvement for the AD8233 output signal (Figure 13b). The signal quality was measured on a scale of 500 mV/Div with a 14-bit Analog Discovery oscilloscope, revealing smooth ECG waves and an almost noise-free trace, which makes this signal usable in a system with an ADC with a resolution greater than 10 bits. This analog signal still needs to pass through the ATmega328 microcontroller that has only a 10-bit ADC, which might set the floor noise of the system. 

**Figure 14:**
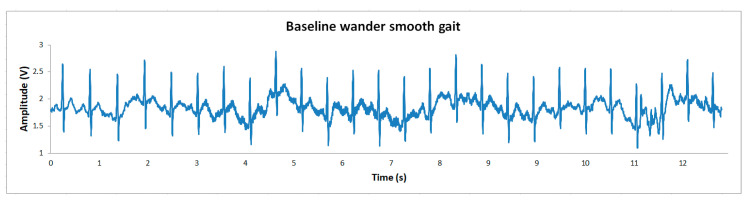
Baseline wander during smooth gate. I-Lead ECG signal from human volunteer to check the baseline wander during a smooth gait of approximately 18 m/min. The signal was transmitted by Bluetooth at 115,200 baud rate and a sampling rate of 500 Hz.

From the multiple prototype tests of real ECGs, considering male volunteers revealed that the peak-to-valley ECG waveform voltage span is 1 V average, which leaves ±1.15 V of excursion for baseline wandering before saturation. The device produces a clean baseline that might wander but is low noise, as is evident from the analog output of the AD8233 in Figure 13b. Another aspect to consider regarding the ECG signal is movement artifacts, which can cause saturation of the signal as the baseline fluctuates. Our application was designed for a resting heart monitor (see Section 2.3), which assumes that the patient remains relatively still during the measurement, but if the patient engages in moderate activity, motion artifacts will appear. A smooth gait test was performed to check the variation of the baseline and to capture the ECG signal. To avoid saturation during exercise, an adjustment must be made to the filter stages to reduce motion artifacts, or the system gain should be decreased to allow greater range before saturation is reached. Figure 14 illustrates the ECG signal baseline wander during a smooth gait without reaching the saturation point. However, due to the analog filters implemented, the movement of the patient during the measurement must be limited. The system can accept a smooth gait, but in this resting heart monitor design the filter stage does not reduce motion artifacts effectively.

With not moving patient measurements, clean ECG waveforms with minimal distortion were obtained. To quantify how clean the ECG was, noise was characterized by the Root Mean Square Error (RMSE) of the baseline trend on smooth segments of the ECG. The smooth segments of the ECG baseline were collected after the end of the T wave and before the onset of the P wave. This simple characterization distinguishes the clean ECG from the noise. See Table 10 for the baseline noise of both AFE breadboard prototypes; the RMSE and range variation over the ECG baseline segments were both calculated. These calculations were made at the analog output of the AFEs and at the full system digital output transmitted. After a gain of 1100 × in the signal path, the AD823X output RMSE indicates the low noise nature of the system, and the RMSE of the digital output demonstrates the added LSB of noise of the 10-bit ADC. It appears now that the AD823X AFE chips require ADCs with 12 bits or better.

**Figure 15:**
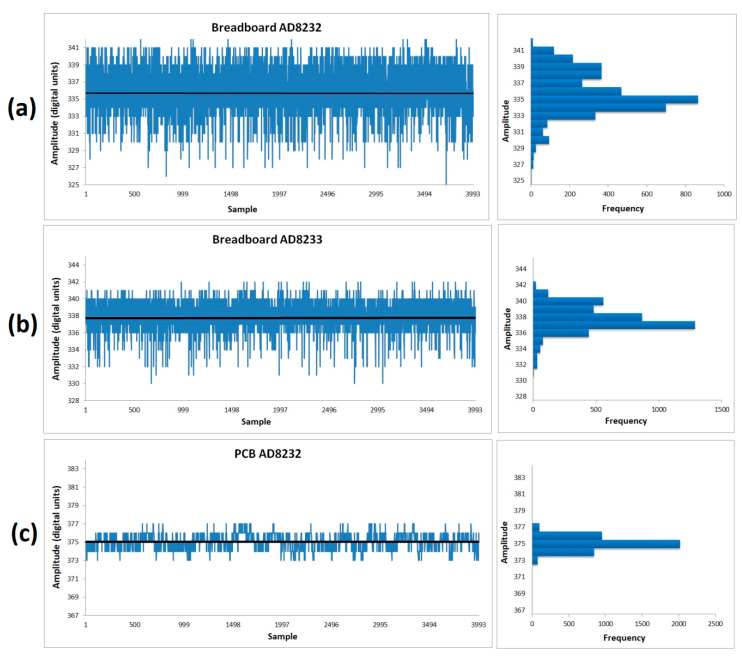
Noise output test when the electrode inputs are tied together. (**a**) Breadboard with the AD8232 AFE microchip; (**b**) Breadboard with the AD8233 AFE microchip; (**c**) PCB with the AD8232 AFE microchip. The left side illustrates the amplitude plots for each sample. The right side displays the statistical histogram of the test.

Part of the calibration of the system consisted of measuring the digital output when a zero-input signal is applied. This process informs us whether it is guaranteed that the combination of all the component circuits has not degraded the stated noise performance of the AD832X microchips. The zero-input signal is created by shorting the +IN and –IN pins of the electrodes and connecting them to virtual ground. Figure 15 illustrates the output signal for the zero-input condition of the three setups tested: Breadboards AD832X and the PCB AD8232 version. Table 11 presents the corresponding RSME and peak-to-peak noise calculations for each device. There is an improvement of noise level from Figure 15a–c, which is corroborated by the data in Table 11. The Breadboard AD8232 had the greatest level of noise, and the PCB AD8232 the lowest. To make this noise data a tool of quality comparison, it is necessary to determine the maximum peak-to-peak noise variation and divide it by the system’s gain (1100), and this value should be similar to the peak-to-peak voltage noise specification of the microchips (of several µVs). This factor appears to be met by the PCB AD8232 version, with only four digital units of maximum peak-to-peak noise variation. Since one LSB digital unit is 4.88 mV; four digital units are 19.52 mV. Dividing this value by AD8232′s gain (1100) results in 17.7 µVp-p, which is close to the stated noise performance of 14 µVp-p. Therefore, the fabricated device did not degrade the original performance of the AD8232 integrated circuit, and we expect that a PCB version with the AD8233 will outperform this, as it has in other tests.

**Figure 16:**
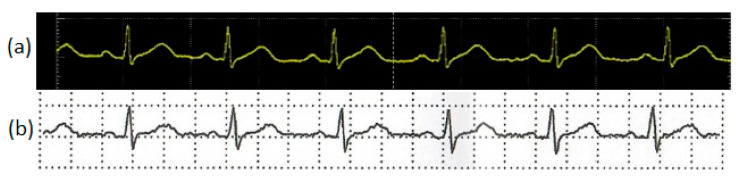
I-Lead ECG signal comparison from human volunteer: (**a**) Upper trace: signal using the AD8232 microchip with oscilloscope hardware: Analog Discovery; and (**b**) Lower trace: medical desktop ECG Mitral M12USB. These ECG signals traces were taken over seven days from the same volunteer.

For the validation of signal quality, a visual comparison with a piece of medical equipment was made. A Mitral M12USB desktop medical electrocardiograph was used as a signal reference, taking Lead-I ECG. Figure 16 illustrates the comparison using the analog signal from the Breadboard AD8232 microchip with the Analog Discovery oscilloscope. These ECG signals were taken over seven days from the same human volunteer. Measurements of the amplitude and duration of the ECG signal validate the coincidence with the range established in the literature as normal. This ECG study helps us to confirm qualitatively that the morphology of the signal captured with our device is comparable to that obtained using a piece of medical ECG equipment.

**Figure 17:**
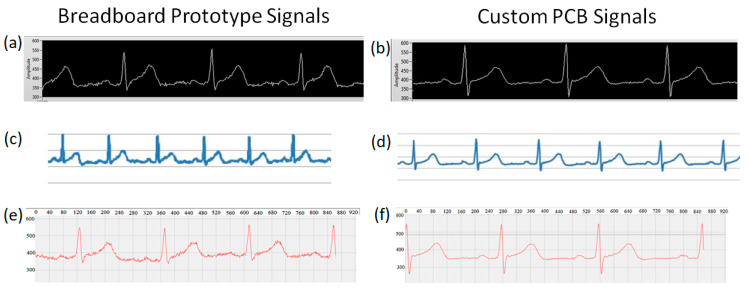
Snapshots of different operating modes with their respective visualization software: (**a**) ECG signal transmitted by serial cable (breadboard prototype); (**b**) ECG signal transmitted by serial cable (custom PCB); (**c**) ECG recorded to a microSD card (breadboard prototype); (**d**) ECG recorded to a microSD card (custom PCB); (**e**) ECG signal transmitted by Bluetooth via smartphone (breadboard prototype); (**f**) ECG signal transmitted by Bluetooth via smartphone (custom PCB). The sampling frequency for all signals was 360 Hz. The ECG signals come from the prototypes using the AD8232 microchip. These signals were all obtained from a human volunteer on the same day.

**Figure 18:**
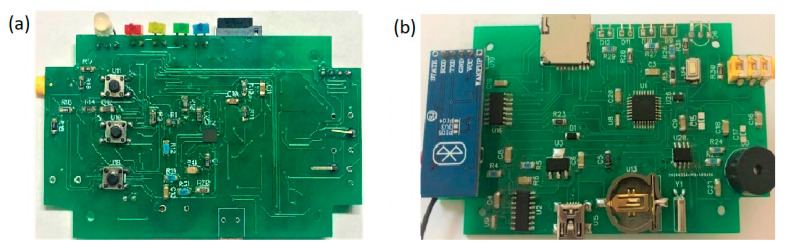
(**a**) Printed circuit board (custom PCB) top view and (**b**) bottom view.

The competence of this Arduino platform regarding applying digital filters was also evaluated. It was determined that the platform has sufficient memory and runtime to apply simple digital filters and to transmit via Bluetooth on Arduino to a smartphone for visualization. This platform was tested using the difference equation *y* [*n*] = *x* [*n*] + *x* [*n* − 3], which rejects the 60 Hz frequency and its harmonic components at a sampling frequency of 360 Hz. To demonstrate the implementation of the prototype breadboard circuit, signals were acquired to evaluate its operation in three modes: serial transmission, microSD recording, and Bluetooth transmission (Figure 17a,c,e). Similarly, to demonstrate the implementation of the custom PCB, new ECG signals were acquired with this printed circuit, and an improvement in the signal quality was noted (see Figure 17b,d,f). The idea in Figure 17 is to demonstrate the quality improvement between the breadboard and the PCB signal. The signals between the different modes of operation were acquired with the same human volunteer. The objective was not to compare the software applications but to reveal the functionality and versatility of the device in the modes of operation, as well as the different display options. The final dimensions of the board are 9.6 × 5.6 cm, as displayed in Figure 18. 

Different levels of data can be stored, viewed, or monitored from ECG records in different operating modes. During serial transmission mode (Mode 1) and Bluetooth transmission modes (Modes 3 and 4), the display is real-time (online), so the possibility of storing data is limited by the size of the data buffer. This data buffer varies depending on the interface on which the data information is displayed or visualized. This factor can be a drawback of these operating modes since it is not possible to retrieve buffers from signals previously sent. For example, in serial transmission mode, the Arduino IDE’s Serial Monitor tool allows the retrieval of a buffer of 450,000 data samples, which, for a 500 Hz sampling rate, can equal about 15 min of a signal. In Mode 2, microSD recording allows the storage of up to 36 h of recording (at a 500 Hz sampling rate) and access of the signal offline for review or processing. Table 12 lists the size of the data buffers in the different modes and interfaces in which the signal is visualized.

A program was executed to corroborate the influence of the class of microSD memory on the write latency. The program fills a buffer with an array of 512 letters (bytes) in the microcontroller’s memory. A time flag begins when starting to write the buffer in the SD memory, another time flag is taken at the end of the writing, and these flags are used to calculate the latency time. It was observed that some aspects, such as the memory brand, influence the write latency, but in general, the Class 10 memory cards had lower write latency (see Table 13).

Using a double buffer corrects the write delay of the microSD card, and although there is RAM available for a third buffer, it was not necessary. Following the double buffer implementation, the ECG recordings were reviewed using automatic analysis methods, and the occurrence of atypical events in all records was eliminated. Recordings of the ECG signal for up to 36 h at the sampling rates of 360 and 500 Hz were fully verified. Thus, by implementing a double buffer to compensate for the write latency in the SD memory, it was possible to avoid overflow and information loss.

The development and validation presented demonstrate the fabrication of a low-cost ECG device with AFE AD823X microchips, and the capabilities and limitations of using open-source hardware and software were exposed. One limitation of the prototype is the rigid analog filter structure (non-programmable); however, the results correspond with our objectives of long-term recording, being portable, rapid design, and signal quality.

## 4. Conclusions

The methodology for the fabrication of an ECG system for long-time personal monitoring using AFE AD823X microchips opens up the possibilities of taking care of CVDs with low-cost devices and illustrates some of the challenges that a researcher faces when developing a technology of this type. The progress of improving the development process was illustrated from breadboard to PCB prototypes and from AD8232 to AD8233 improvements. The results indicate that the AD8232 and AD8233 microchips are suitable for the AFE function, as they delivered a useful signal for a long-term single-lead ECG monitoring application. However, the AD8233 microchip demonstrated higher signal quality, although it was only evaluated in the breadboard circuit; therefore, there remains potential performance improvement when the new PCB is manufactured with AD8233. The ATmega328 microcontroller on the Arduino open-source platform also provided satisfactory results. Typical microcontrollers in these popular platforms have a 10–12 bits ADC, but the achieved ECG signal quality in this study realized the potential use of high-precision microcontrollers with ADCs of 16 bits or greater. The flexible recorder presented in this paper can work for high-resolution electrocardiography (HRECG) and other biopotential measurement applications in which the minimum sampling frequencies are ≥ 1 KHz. While our device was set for a specific bandwidth for testing, the results obtained demonstrate that it can accommodate these high-resolution applications also. Furthermore, a narrower band-pass filter, and lower gain combined with high resolution ADC (20 bits or more, to maintain signal quality) will tolerate motion artifact conditions. Therefore, once a specific application is decided and bounded, optimizing low-power microcontroller techniques such as “sleep mode” would be good idea to implement to reduce power consumption, since work in low-power requirements for a mobile monitoring device is still required. With its various communication protocols, the microcontroller kept the fabrication cost low, maintained portability, and reduced the number of components and the design time of the prototype. The development and experimentation presented expose the capabilities and limitations of using open-source hardware and software. Using generic Arduino boards and Bboards because of their reduced price, the total cost for single prototype components was 20 USD, excluding the cost of the battery, the microSD memory, and PCB manufacturing. This price renders a personal monitoring ECG system with prolonged recording time accessible to a larger sector of the population. This design does not seek to replace hospital equipment but can assist the diagnosis, prevention, and management of CVDs. Moreover, it is not a completed system design yet; additional effort must be made to ensure compliance with medical safety guidelines from regulatory agencies. The high-quality digitized ECG signal reveals the potential for clinical grade applications, not only simple monitoring, in the home environment instead of expensive healthcare facilities. Finally, one immediate future of this modular technology is the possibility of cloud storage.

## Figures and Tables

**Table 1:** Characteristics of modern mobile health electrocardiographs on the market.

ECG Model/Name	Channel	CMRR (dB)	Bandwidth	Sampling Rate	Recording Time	Cost (USD)	User Interface	Data Storage
**Iwatch apple** [6]	Single channel	N/A	N/A	N/A	30 s	499–699	IPhone, Ipad	Internal device memory
**Alivecor, Kardia** [7]	Single channel	76	0.5–40 Hz	300 Hz	30 s	180	Smartphone	Internal device memory
**CardioSecur** [8]	Single channel	N/A	0.5–40 Hz.	250–500 Hz	30 min	130 + 120 subscription	LCD screen and software	MicroSD card
**Qardiocore** [9]	Single channel	N/A	0.05–40 Hz	600 Hz	24 h	586	Smartphone	Internal device memory
**ECG Anywhere** [10]	Single channel	105	N/A	500 Hz	N/A	202	Smartphone and tablet	Internal device memory
**Spyder wireless ECG** [11]	Single channel	100	N/A	125 Hz	72 h	500	Smartphone	Internet cloud service
**Silmee** [12]	Single channel	N/A	N/A	1 K Hz	24 h	880	PC software	MicroSD card.
**Zio patch** [13]	Single channel	N/A	0.5–30 Hz	200 Hz	336 h single use	1050	Software and interpretation service online	Internal device memory

N/A: not available.

**Table 2:** Tracing the connections for the ECG prototype sections.

Electrode	AFE AD8232	AFE AD8232	Data Logger Shield	Arduino Nano	BT HC-05	Arduino Nano	Discrete Component	Other GND Connections
Left arm LA	+IN pin 2 through 180K resistance	output signal, pin 10	CS, pin 8	D8, pin 11	Tx, pin 4	A2, pin 24	Transmit LED anode	All LED cathodes
Right arm RA	−IN pin 3 through 180K resistance	Vs, pin 17	MOSI, pin 7	D9, pin 12	Rx, pin 5	A1, pin 25	Stop LED anode	All Button GND side
Right Leg RL	RLD pin 5 through 360K resistance	GND, pin 16	MISO, pin 6	5V, pin 27	Vcc, pin 2	VIN, pin 30	(+) Battery liPO 7.4V	
		D13, pin 16	SCK, pin 5	GND, pin 4, 29	GND, pin 3	GND, pin 4, 29	(−) Battery liPO 7.4V	
		A5, pin 21	SCL, pin 4			D4, pin 7	Red LED anode	
		A4, pin 22	SDA, pin 3			D3, pin 6	Yellow LED anode	
		GND, pin 4, 29	GND, pin 2			D2, pin 5	Green LED anode	
		5V, pin 27	VCC, pin 1			A6, pin 20	Blue LED anode	
						D7, pin 10	Button Stop	
						D6, pin 9	Button Mode Selection	
						D5, pin 8	Button Transmit/record	

**Table 3:** Structure of design of experiments. Once the three factors are identified (Figure 11), they are placed in a table that expresses the possible combinations between them. The identified factors are as follows: (A) function generator, (B) SD card memory type, (C) error per data logger shield. For the purpose of this experiment, it was agreed that three repetitions of each combination were sufficient. In the cells marked with “x,” the number of blackout events must be placed, being the output variable.

**Factor B: (Memory Type)**	**Factor A: Function Generator Type**			
	A1. Digilent Generator		A2. Tektronik Generator	
	**Factor C: Data Shield**		**Factor C: Data Shield**	
	C1. Data Logger Shield	C2. SD and RTC Shield	C1. Data Logger Shield	C2. SD and RTC Shield
B1. Low-speed memory (class 2)	x	x	x	x
	x	x	x	x
	x	x	x	x
B2. Medium-speed memory (class 4)	x	x	x	x
	x	x	x	x
	x	x	x	x
B3. High-speed memory (class 10)	x	x	x	x
	x	x	x	x
	x	x	x	x
(x) Output variable: Number of blackout events.				
**Factors that can affect the output variable.**				
Factor A: Function generator	A1. Analog discovery (Digilent)			
	A2. AFG3021B (Tektronik)			
Factor B: MicroSD memory	B1. Low-speed memory (class 2)			
	B2. Medium-speed memory (class 4)			
	B3. High-speed memory (class 10)			
Factor C: Data shield	C1. Data logger shield			
	C2. MicroSD and RTC shield			

**Table 4:** Data extracted from the experiment. Output variable: number of blackout events; Factor A: generator type; Factor B: memory class; Factor C: data logger module.

**Factor B: (Memory Type)**	**Factor A: Function Generator Type**			
	A1. Digilent Generator		A2. Tektronik Generator	
	**Factor C: Data Shield**		**Factor C: Data Shield**	
	C1. Data Logger Shield	C2. SD and RTC Shield	C1. Data Logger Shield	C2. SD and RTC Shield
B1. Low-speed memory (class 2)	4	5	11	9
	4	7	8	8
	3	8	10	4
B2. Medium-speed memory (class 4)	6	6	5	6
	6	6	6	6
	6	7	6	4
B3. High-speed memory (class 10)	4	2	1	2
	1	3	1	3
	2	1	5	3

**Table 5:** ANOVA study results. A *p*-value < 0.05 means that the factor or interaction has a significant effect on the output variable. Only Factor B complies with *p*-value < 0.05 corresponding to the type of SD card memory.

Source	DF	Sec SS	Adj SS	Adj MS	F-Value	*p*-Value	*p* < 0.05	Effect
FACTOR A	1	3.361	3.361	3.361	2.57	0.122	accepted	No
FACTOR B	2	68.167	68.167	34.083	26.11	0.000	rejected	Yes
FACTOR C	1	0.250	0.250	0.250	0.19	0.666	accepted	No
FACTOR A*FACTOR B	2	7.389	7.389	3.694	2.83	0.079	accepted	No
FACTOR A*FACTOR C	1	0.028	0.028	0.028	0.02	0.885	accepted	No
FACTOR B*FACTOR C	2	1.167	1.167	0.583	0.45	0.645	accepted	No
FACTOR A*FACTOR B*FACTOR C	2	1.056	1.056	0.528	0.40	0.672	accepted	No
Error	24	31.333	31.333	1.306				
Total	35	112.750						

**Table 6:** Offline algorithms for ECG analysis.

Algorithm Name	Platform	Commercial	Long-Term Files (24 h)
Biomedical Workbench [36]	LabVIEW	Yes	Visualization and Manipulation
BioSigKit [37]	Windows (MATLAB)	No	Visualization
QRS Detection [38]	Windows (MATLAB)	No	Not supported
Pan-Tompkins Algorithm [39]	Windows (MATLAB)	No	Visualization and Manipulation
Simple RTS [40]	Windows (MATLAB)	No	Not supported

**Table 7:** Software and algorithm evaluation for automatic ECG signal analysis. The control signal is created with a specific number of QRS peaks.

Archive		MATLAB								LABVIEW	
	Control Signal	BioSigKit		Pan-Tompkins		nQRS Detector		Simple QRS		Biomedical Workbech	
		Detected	Error	Detected	Error	Detected	Error	Detected	Error	Detected	Error
1-h without blackout events	3600	3600	0	3600	0	3600	0	3601	1	3600	0
1-h with 5 blackout events	3595	3596	1	3595	0	3599	4	3598	3	3595	0
2-h without blackout events	7200	7200	0	7200	0	7200	0	7201	1	7200	0
2-h with 5 blackout events	7195	7196	1	7195	0	7199	4	7198	3	7195	0

**Table 8:** ECG breadboard prototype specifications.

Parameter	ECG Prototype
Application	Monitoring
Bandwidth	0.5–40 Hz
Gain	1100
Sampling frequency	360–2380 Hz
Dynamic range (peak to valley)	3 mV
Patient leakage current	1–2 µA
CMRR	88.7 dB
Input impedance	10 GΩ
Noise	17.7 µV
Offset DC	±300 mV
Recording time	36 h
Components accuracy	1%

**Table 9:** Maximum data/second to avoid information loss in different operating modes.

Operating Mode		Bauds Speed/Clock (Hz)	Interrupt Interval Configured	Maximum Number of Samples/Second (Transferred by Interruption Method)
Mode 1: USB Serial Port to PC (Hardware, Not Modifiable).		230,400	480 µs	2084
		1 MB	420 µs	2380
Mode 2 Recording on SD card (SPI).		set to SPI_HALF_SPEED = 4 MHz	485 µs	2019
Mode 3: Bluetooth to Android phone Bluetooth Graphics application. Bluetooth V2.0+EDR	SoftwareSerial library	115,200	1100 µs	909
	Arduino hardware serial port (pins 0,1)	115,200	800 µs	1250
Mode 4: Bluetooth to IDE serial PC monitor. Bluetooth V2.0+EDR	SoftwareSerial library	115,200	1100 µs	909
	Arduino hardware serial port (pins 0,1)	115,200	800 µs	1250

**Table 10:** Baseline noise of breadboard prototype. RMSE and range variation over ECG baseline reference.

AFE	AD823X Output ^1^		Digital Output ^2^	
	RMSE (mV)	Range ^3^ (mv)	RMSE (mV)	Range ^3^ (mV)
AD8232	8.10	46.65	12.46	53.76
AD8233	3.05	23.38	8.50	50.10

^1^ Taken at pin OUT, connected to the input of the ADC, using the Digilent’s Analog Discovery 2 instrumentation system (@1.5 Ksps). ^2^ Through the Atmega328 microcontroller ADC (@500 sps), by serial communication and Bluetooth HC-05 transmission. ^3^ Range: Difference between maximum and minimum value of the baseline series.

**Table 11:** RMSE and peak-to-peak variation of the digital output when shorting the +IN and –IN electro inputs pins.

Prototype Version	RSME (Digital Units)	Maximum Peak-to-Peak Noise Variation (Digital Units)
Breadboard AD8232	2.6342	17
Breadboard AD8233	1.6701	12
PCB AD8232	0.7936	4

As one LSB digital unit = 4.88 mV, four digital units = 19.52 mV.

**Table 12:** Data buffer size in each mode of operating/interface for visualization.

Operation Mode	Data Buffer Size
Serial Monitor	450,000 samples
Serial Plotter	500 samples
LabVIEW	1023 samples
Bluetooth Graphics	1680 samples
MicroSD recording	36 h, recording @ 500 Hz

**Table 13:** Write latency measurement in Classes 2, 4, and 10 microSD memory cards.

Capacity (GB)	Brand	Class	Write Latency (µs)
1	Sandisk	4	5312
2	Nokia	2	6312
2	N/A	2	6840
4	Sandisk	4	8240
4	Kingston	10	4584
16	Sandisk ultra	10	5312
32	Sandisk	4	6464
